# Secondary Metabolites of *Osmanthus fragrans*: Metabolism and Medicinal Value

**DOI:** 10.3389/fphar.2022.922204

**Published:** 2022-07-18

**Authors:** Chen-Chen Fu, Fa-Ying Xu, Yu-Chen Qian, Hoi-Lun Koo, Yi-Fan Duan, Geng-Min Weng, Tai-Ping Fan, Mo-Xian Chen, Fu-Yuan Zhu

**Affiliations:** ^1^ Co-Innovation Center for Sustainable Forestry in Southern China and Key Laboratory of National Forestry and Grassland Administration on Subtropical Forest Biodiversity Conservation, College of Biology and the Environment, Nanjing Forestry University, Nanjing, China; ^2^ International Cultivar Registration Center for Osmanthus, College of Biology and the Environment, Nanjing Forestry University, Nanjing, China; ^3^ Department of Chinese Medicine, The University of Hong Kong-Shenzhen Hospital, Shenzhen, China; ^4^ RCI Research Institute Limited, Hong Kong, China; ^5^ Department of Pharmacology, University of Cambridge, Cambridge, United Kingdom

**Keywords:** *Osmanthus fragrans*, secondary metabolites, medicinal compound, terpenoids, flavonoids, phenolic acids

## Abstract

*Osmanthus fragrans* (scientific name: *Osmanthus fragrans* (Thunb.) Lour.) is a species of the *Osmanthus* genus in the family Oleaceae, and it has a long history of cultivation in China. *O. fragrans* is edible and is well known for conferring a natural fragrance to desserts. This flowering plant has long been cultivated for ornamental purposes. Most contemporary literature related to *O. fragrans* focuses on its edible value and new species discovery, but the functional use of *O. fragrans* is often neglected. *O, fragrans* has many properties that are beneficial to human health, and its roots, stems, leaves, flowers and fruits have medicinal value. These characteristics are recorded in the classics of traditional Chinese medicine. Studies on the metabolites and medicinal value of *O. fragrans* published in recent years were used in this study to evaluate the medicinal value of *O. fragrans*. Using keywords such as metabolites and *Osmanthus fragrans*, a systematic and nonexhaustive search of articles, papers and books related to the medicinal use of *Osmanthus fragrans* metabolites was conducted. Fifteen metabolites were identified through this literature search and classified into three categories according to their properties and structure: flavonoids, terpenes and phenolic acids. It was found that the pharmacological activities of these secondary metabolites mainly include antioxidant, anticancer, anti-inflammatory and antibacterial activities and that these metabolites can be used to treat many human diseases, such as cancer, skin diseases, cardiovascular diseases, and neurological diseases. Most of the reports that are currently available and concern the secondary metabolites of *Osmanthus fragrans* have limitations. Some reports introduce only the general classification of compounds in *Osmanthus fragrans*, and some reports introduce only a single compound. In contrast, the introduction section of this paper includes both the category and the functional value of each compound. While reviewing the data for this study, the authors found that the specific action sites of these compounds and their mechanisms of action in plants are relatively weak, and in the future, additional research should be conducted to investigate this topic further.

## Introduction

### Introduction to *Osmanthus fragrans*

*O. fragrans* is widely cultivated in China and was documented in the ancient pharmacopeia. During the Ming Dynasty (AD 1368–1644), the medicinal functions of *O. fragrans* were documented in Li Shizhen’s monumental pharmaceutical encyclopaedia Compendium of Materia Medica*. O. fragrans* is classified as a pungent, warm and nonpoisonous Chinese herb. It is used to promote saliva secretion, deodorize and clear phlegm. It is believed to be effective in the treatment of toothaches. Descriptions of the uses of *O. fragrans*, such as “*O. fragrans* root can be used to treat toothache due to virtual fire,” “*O. fragrans* flower can be used to treat bad breath” and “*O. fragrans* fresh bark or root bark is applied to the injured area to treat sprains” are found in the Compendium of Materia Medica. In recent years, modern scientific and technological strategies have been used to further study *O. fragrans*. For example, it has been found that the essential oil of *O. fragrans*, which is a volatile component, mostly contains terpenoids, as well as flavonoids and other glycosides, pigments, etc., which have anticancer, antioxidant, anti-inflammatory, analgesic, and cardiovascular effects (Li and Huang, 2020; Cheng, 2021). There are currently approximately one hundred varieties of *O. fragrans*, which can be divided into four categories, namely, the *Luteus* group, *Albus* group, *Aurantiacus* group, and *Asiatiacus* group, according to their traits and characteristics. *O. fragrans* is an evergreen shrub in the Oleaceae family. It is a deciduous tree with flowers that grow in small panicles. *O. fragrans* flowers can be produced in the spring, summer and autumn, but its peak flowering time is usually approximately September to early October. *O. fragrans* has the nickname “Jiulixiang,” which literally means “nine Chinese miles fragrant” because of its far-reaching scent. This property makes *O. fragrans* a common choice for achieving a strong and long-lasting fragrance in foods (dessert, pastry, tea and wine) as well as in producing perfumes and cosmetics. It has been reported in the literature that the terpenoids in the leaves of *O. fragrans* can inhibit the production of melanin (Yang, et al., 2021). Excessive deposition of melanin in the human body can lead to various skin diseases; thus, *O. fragrans* is used in the cosmetic industry to whiten the skin and alleviate skin diseases (Le, et al., 2022).

The medicinal uses of *O. fragrans* vary depending on the metabolite content of the plant. The flavonoid synthesis and carotenoid synthesis pathways are similar in the four categories of *O. fragrans*. However, due to variations in floral fragrance, flower colour, and genetic characteristics, the varieties in each category exhibit slight differences in metabolic pathways. Therefore, the types and quantities of anabolic metabolites in these different varieties are not the same. In terms of floral fragrance, α-ionone and β-ionone are the most important aroma-producing substances in *O. fragrans*. As the most aromatic varieties, varieties in the *Albus* group possesses the highest levels of α- and β-ionone, followed by those in the *Asiatiacus* group, those in the *Aurantiacus* group and those in the *Luteus* group. The varieties in the *Luteus* and *Asiatiacus* groups are sweet and greasy, and those in the *Aurantiacus* group have the weakest aroma among those in all the groups.

α-Carotene and β-carotene play a major role in determining the flower colour of *O. fragrans*. Varieties in the *Aurantiacus* group contain both α-carotene and β-carotene, making them orange–red. Varieties in the *Luteus* group conta only one (α- or β-carotene). Varieties in the *Asiatiacus* group may contain α-carotene or β-carotene, but those in the *Albus* group do not. Therefore, varieties in the *Luteus* group are mostly yellow, those in the *Albus* group are mostly light yellow, those in the *Aurantiacus* group are mostly orange–red, and those in the *Asiatiacus* group are between the colour of varieties in the *Luteus* and those in the *Albus* group, with a slightly greater similarity towards the colour of those in the *Luteus* group (Wang, 2009; Dan, 2014). Therefore, in most cases, we can distinguish *O. fragrans* varieties by flower colour.

### Medicinal Value of *O. fragrans* Metabolites

**FIGURE 1:**
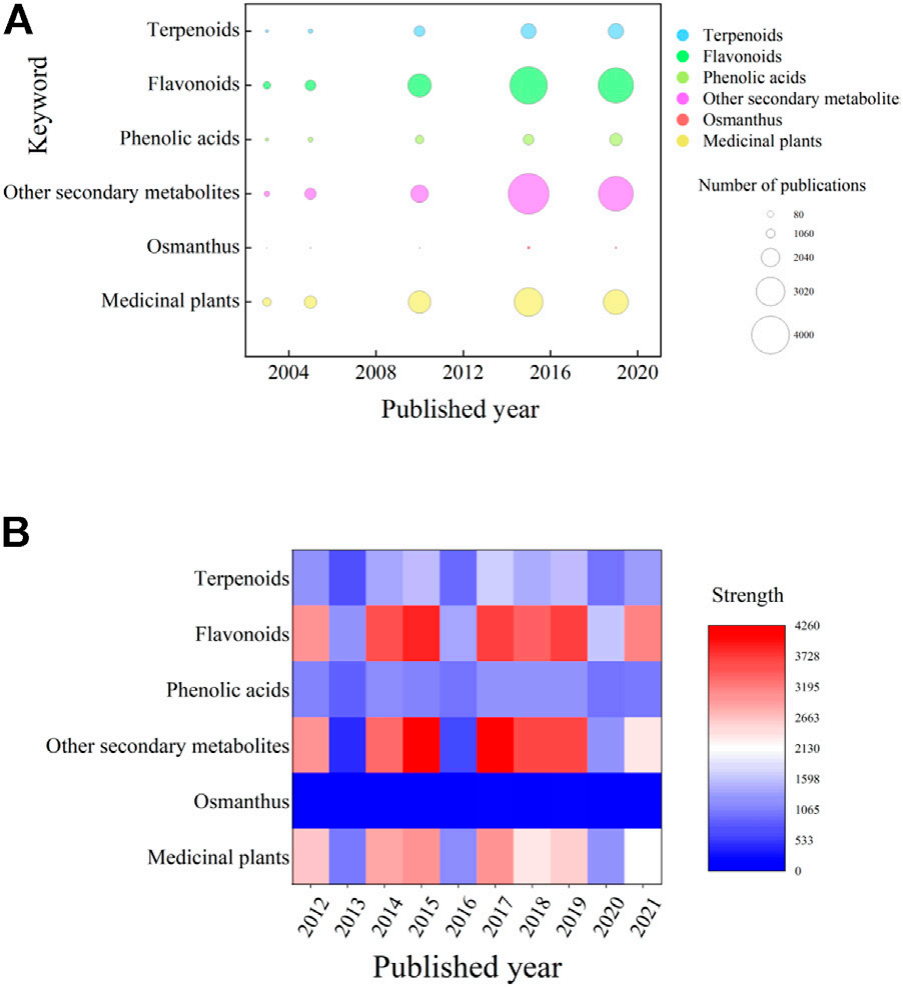
Summary of the literature according to keywork and the year published over the last 10 years. **(A)** Bubble chart showing the number of publications in 5 years from the past 10 years. The horizontal axis is the publication year, and the vertical axis is the relevant keyword used to search the literature. Correlative studies are relatively weak, and studies of various secondary metabolites are clearly differentiated. **(B)** Heatmap showing the prevalence of keywords in publications over the past 10 years. The figure shows that the research statuses of the three major categories of secondary metabolites selected in this paper are very different, with research on flavonoids being the most extensive and research on phenolic acids being the least extensive. (The basic principle of the author’s preliminary background investigation before writing this manuscript is to first conduct a general search on the research progress of *O. fragrans*, select compounds extracted from *O. fragrans*, and then screen out relatively important secondary metabolites of *O. fragrans*).

There are many metabolism pathways that occur throughout the lifespan in *O. fragrans*, and these pathways lead to the generation of many secondary metabolites. These secondary metabolites have some degree of medicinal value; however, there have been few studies on their pharmacological mechanisms. In recent years, metabolomics has become an increasingly popular technique for performing more vigorous research on medicinal plants. Metabolomic studies have shown that the roots, stems, leaves, flowers, and fruits of *O. fragrans* have medicinal properties. These characteristics were recorded in the classics of traditional Chinese medicine. *O. fragrans* roots can be used to treat rheumatic numbness and low back pain. *O. fragrans* flower extract can also be used to treat bad breath (Wu, et al., 2017; Li et al., 2020c). The extraction methods used for different parts of *O. fragrans* are also different. An ultrasonic-assisted extraction method, during which the seeds are peeled, dried in a drying oven, and finally pulverized into powder for use, is used for extraction of *O. fragrans* seeds. Fresh *O. fragrans* is often extracted by the ethanol reflux method. Like seeds, fresh *O. fragrans* is first dried, crushed, and finally stored (Li et al., 2020b; Ning, et al., 2020). Liao et al. used HPLC–MS to analyse the biological activity of *O. fragrans* root and identified dozens of compounds in *O. fragrans* root, including the first reported compound. The results of network pharmacology showed that *O. fragrans* root is effective in the treatment of inflammation, cardiovascular disease, cancer and rheumatoid arthritis, which is consistent with traditional claims of the medicinal value of *O. fragrans* root (Liao, et al., 2021). In the latest reports on *O. fragrans*, many new compounds have been reported, and *O. fragrans* extracts have been shown to have a variety of biological activities *in vitro* and *in vivo*. *O. fragrans* extract is rich in flavonoids [dihydroquercetin (DHQ), luteolin, naringenin, etc.), phenolic acids (tyrosol (Ty), rosmarinic acid (RA), protocatechualdehyde (PCA), etc.], terpenoids (loganic acid, oleanolic acid, maslinic acid, etc.), glycosides (salidroside, salidroside, etc.), fatty acids (oleic acid, linoleic acid, palmitic acid, etc.), pigments (melanin, red pigment, etc.) and many other active ingredients, and *O. fragrans* is the plant that contains the most flavonoids (Song et al., 2021a; Wang, et al., 2022). To evaluate the medicinal value of *O. fragrans*, 15 natural metabolites with biological activity were identified according to the metabolic pathways in *O. fragrans* through a search of studies on the synthetic pathways and chemical structures of the metabolites (Figure 1). These 15 *O. fragrans* metabolites can be classified into three categories based on their structure: 1) flavonoids (diosmetin (DIO), DHQ, astilbin, luteolin, and naringenin), 2) terpenoids (loganic acid, oleoside, secoxyloganin, oleanolic acid, and maslinic acid), and 3) phenolic acids (Ty, p-hydroxyphenylacetic acid, RA, salicylic acid (SA), and PCA) (Wu, et al., 2017; Yang, et al., 2021). Although some of these metabolites are not unique to *O. fragrans*, their medicinal effects are still of research value and may shed light on their therapeutic value. Recently, a review article published by Wang et al. introduces *O. fragrans* in several aspects, including botanical description, geographic distribution, traditional uses, phytochemical compositions, and pharmacological properties. However, the molecular mechanisms of these active metabolites remain unclassified. To better understand the medicinal value of secondary metabolites in *O. fragrans*, this article selected 15 representative secondary metabolites and introduced them from the molecular level: 1) to study their potential synthetic pathways in *O. fragrans* and regulatory mechanisms, 2) to understand their related physicochemical properties and synthetic pathways, 3) to study how they can treat related diseases through signal transduction (Table 1).

**TABLE 1:** Fifteen major secondary metabolites in *O. fragrans*.

Small molecule	Structure	Chemical properties	Mechanism and pathways	Related experimental information	References
Flavonoids					
Diosmetin	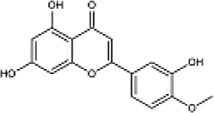	Yellow powder	Exerts anti-inflammatory, antibacterial, and antioxidant effects; affect the activation of thePI3K/Akt and NF-κB signalling pathways	The cardiomyocyte cell line H9c2 derived from the rat; Dosages: 5, 10, or 15 μg/ml; cells were incubated in normoxia for 1 h, then in hypoxia for 48 h	(Chen, et al., 2020a) (Si, et al., 2020)
Dihydroquercetin	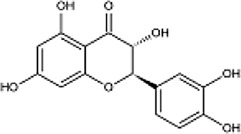	Pale yellow or colorless needle-like crystals	Protect the kidneys. Affect the mTORC2/Akt signalling pathway	65 adult male SD rats weighing 220–240 g; treatment doses of 50 mg/kg, 100 mg/kg, 200 mg/kg for 12 weeks; Urine is collected and blood drawn after experimental treatment	Qiao, et al. (2020)
Astilbin	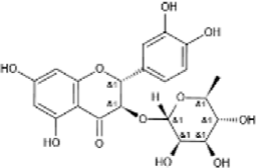	White crystalline powder	Protect the nervous system.Affect the PI3K/Akt and MAPK pathways	20 adult male rats; 50 mg kg ^−1^ pretreated for 2 h. Analysis after 24 h of reperfusion C57BL/6 mice	Yu, (2020)
Luteolin	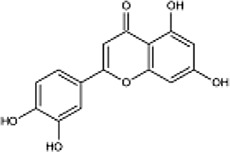	Yellow needle-like crystals	Anti-gout, exerts antitumour effects; enhances memory. Inhibits the production of the inflammatory factors TNF-α and IL-1β	50 ICR mice were treated for about 7 days, dosage: 20, 40, 80 mg/kg	Yue, (2019)
Naringenin	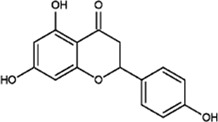	Yellow powder	Protects against lung damage and ageing; Neuroprotection. Regulate the PKC, Akt, MAPK and other signalling pathways	Well differentiated PC12 cells; diluted gradients of 400, 40, 4, 0.4, 0.04, 4 × 10^–3^, 4 × 10^–4^, 4 × 10^–5^ μmol L^−1^ were used, 5 days	(Qi, et al., 2020) (Wang and Wang, 2021) (Zeng, et al., 2018)
Terpenoids					
Loganic acid	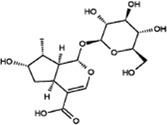	White crystalline powder	Exerts anti-lipogenesis and anti-osteoporosis effects. Reduce the expression of key adipogenesis-related genes such as adiponectin, lipoprotein lipase	40 seven-week-old female OVX mice; doses: 2, 10 and 50 μg/ml. Oral for 12 weeks; Osteoblast MC3T3-E1 cells; dosage: 2, 10 and 50 μM	(Park, et al., 2018) (Park, et al., 2020)
Oleoside	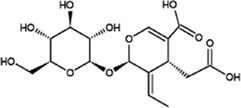	Powder	Exerts antioxidative effects. Act as a free radical scavenging antioxidant and inhibits lipid peroxidation	Cell line HepG2; Dosage: 10^–3^, 10^–4^, 10^–5^, 10^–6^, 10^–7^ mol L^−1^; 36 h	(Fei, et al., 2010) (Araújo, et al., 2021)
Secoxyloganin	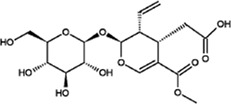	Solid	Anti-virus, antitumor	Human breast cancer (MCF-7) and prostate cancer (PC-3) cell lines; Dosage: 10 μL; 24 h; 140 male guinea pigs weighing 300 ± 50 g	(Camargo, et al., 2020) (Lv, 2021) (Wang, et al., 2016)
Oleanolic acid	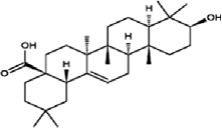	White needle-like crystals	Exerts anti-inflammatory effects, lowers blood lipid. Affects the JNK signalling pathway	Human cell line Eca109; Dosage: 10, 20, 40 μmol L^−1^ 24 h	(Li et al., 2021b) (Zhang, et al., 2021)
Maslinic acid	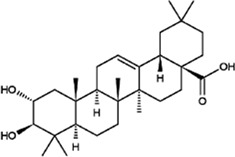	Light yellow powder	Cellular oxidative damage protection. Influence the p38 and Nrf2/HO-1 pathways	Normal rat liver cell line (BRL-3A) Dosage: 5, 10, 15, 20, 25, 30 μmol/L; 8 h	Li Y, et al. (2020)
Phenolic acids					
Tyrosol	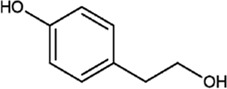	Yellow-green fine powder	Prevents coronary heart disease, promote endothelial cell proliferation	Human microvascular endothelial cells (HMEC-1), Dosage: 7.5, 10, 15, 30, 40, 80 μM; 48 h	Shi, et al. (2020)
p-Hydroxyphenyl acetic acid	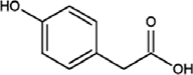	White crystalline powder	Neuroprotection. As a raw material for the synthesis of various drugs	Weigh 10 g (65.72 mmol) of p-Hydroxyphenyl acetic acid, dissolve it with methanol (100 ml)	Hu, et al. (2020)
Rosmarinic acid	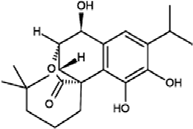	Light yellow powder, white powder	Antiproliferative effect on human melanoma A375 cells. Interference with MAPK/ERK pathway leads to apoptosis	Human melanoma A375 cells, dosage: 1:120, 1:240, 1:480 and 1:960 dilutions; 72 h	Cattaneo, et al. (2015)
Salicylic acid	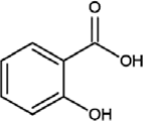	White needle-like crystal or hairy crystalline powder	Treatment of plantar warts, treat skin diseases	240 patients 12 years and older; dosage: 50%; 6 months; 90 patients with melasma; dosage: 20%; 12 weeks	(Cockayne, et al., 2011) (Dayal, et al., 2020)
Protocatechualdehyde	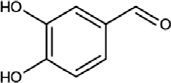	Light beige needle crystals or off-white powder	Neuroprotective effect, promote bone healing. Down-regulation of GFAP and AQP-4 proteins in brain tissue	48 male rats of 240–270 g; dosage: 10, 20 mg/kg; once a day for 5 consecutive days	(Jin, et al., 2021) (Zhang, et al., 2013)

The table introduces the structures and chemical properties of fifteen major secondary metabolites and briefly summarizes their medicinal properties and mechanisms. These properties include the ability to exert antioxidative, anti-ageing, neuroprotective, and antidepressant effects; lower blood lipid and blood sugar levels; and other properties. Relevant experimental information is provided.

## Classification and Overview of Secondary Metabolites

### Flavonoids

**FIGURE 2:**
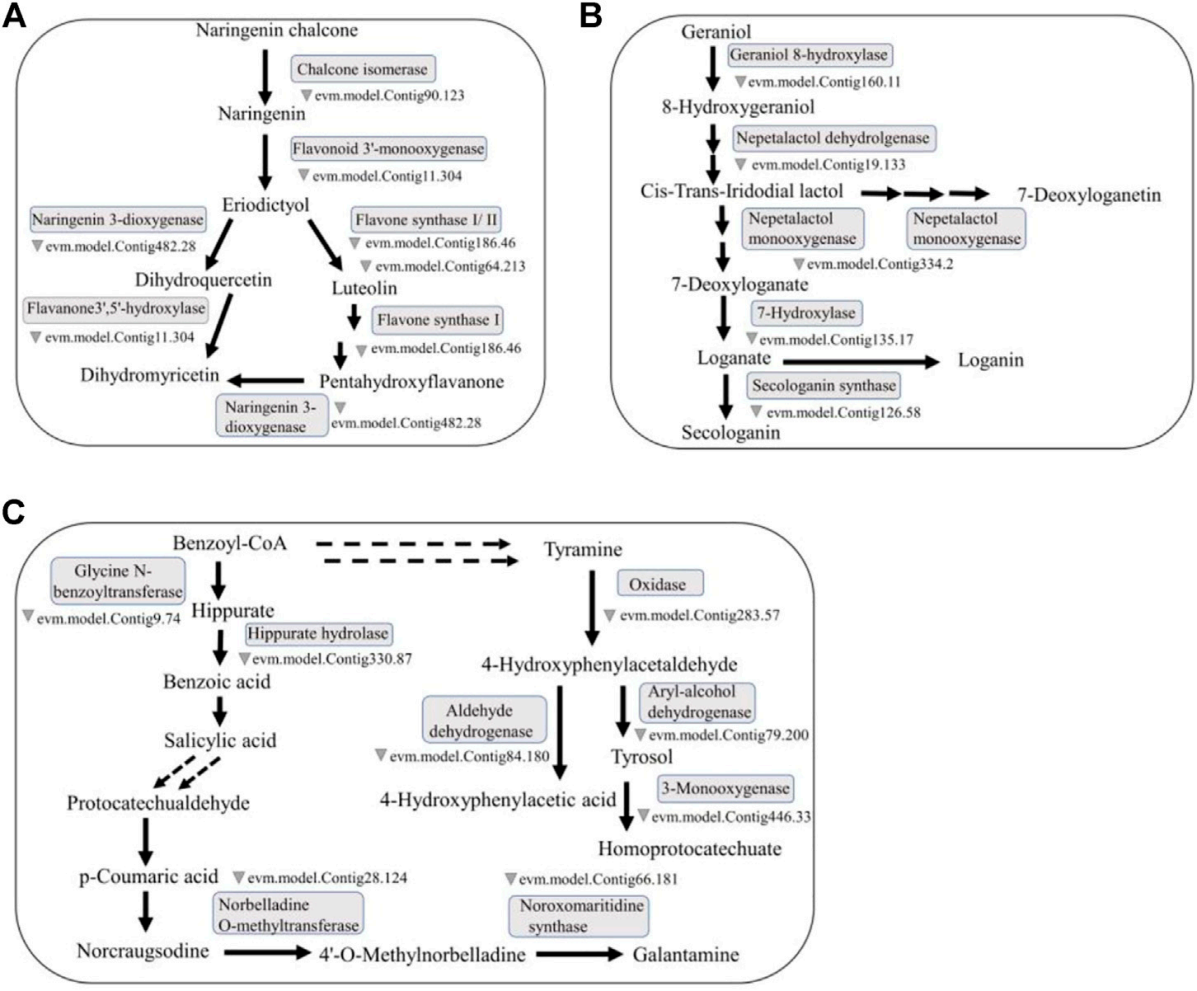
Representative medicinal compounds in *O. fragrans* and their biosynthetic pathways. **(A)** The main synthesis pathway containing naringenin, luteolin and DHQ in the flavonoid biosynthesis pathway is presented. Naringenin can produce luteolin and DHQ through reactions catalysed by different enzymes. **(B)** Loganic acid is a terpenoid. The synthetic pathway of loganic acid in the monoterpene biosynthesis pathway. Loganic acid can be produced from geraniol through reactions catalysed by a series of enzymes. **(C)** SA, PCA and Ty are synthesized via three different phenolic acid biosynthesis pathways, but the synthetic pathway of SA can be connected to the synthetic pathways of PCA and Ty. The double dotted arrow in the figure represents the connection between the pathways. (The relevant *O. fragrans* gene information in this biosynthetic pathway map comes from an article about *O. fragrans* published by) (Yang, et al., 2018).

Flavonoids are an important type of secondary metabolite in *O. fragrans*. By assessing the iron ion reduction ability of the flavonoids in *O. fragrans* and by using the two most commonly used antioxidant assays (the DPPH and ABTS assays), Bao and others showed that these flavonoids show good antioxidant activity (Bao, et al., 2018). Flavonoids are a class of natural compounds with low toxicity and few side effects, which make them more preferable drug candidates than other secondary metabolites of *O. fragrans*. Therefore, research on flavonoids may help determine the potential side effects of drugs containing these metabolites (Figure 2A).

#### Diosmetin

**FIGURE 3:**
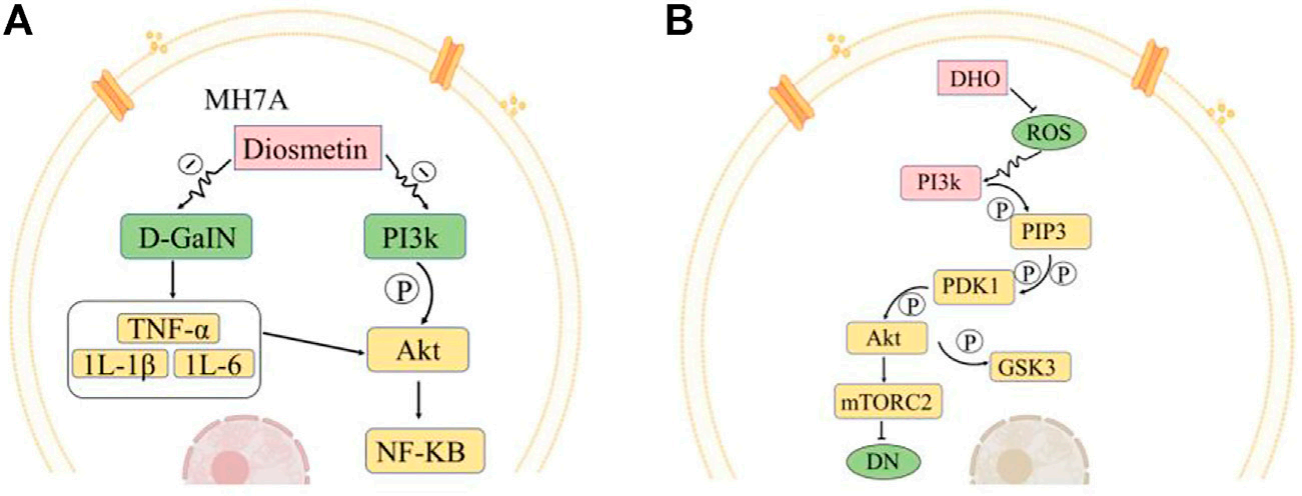
Representative medicinal compounds in *O. fragrans* and their pharmacological mechanisms of action. **(A)** Pathways by which Dio affects MH7A cell apoptosis. Dio inhibits the production of inflammatory mediators and cytokines (TNF-α, IL-1β, IL-6, etc.) induced by D-GalN and can also regulate the Akt/NF-kB pathway. Dio can also inhibit the PI3K/Akt pathway. PI3k is phosphorylated to generate Akt, and Akt is a key molecule in the activation of the NF-κB signalling pathway. Inhibition of this pathway reduces cell proliferation and inflammatory cytokine production in MH7A cells and promotes apoptosis. **(B)** Effects of DHQ on the mTORC2/Akt signalling pathway under high-glucose conditions. Reactive oxygen species (ROS) can activate the protein kinase PI3K signalling pathway. There are two downstream pathways of PI3k. In the one presented here, the PI3k-Akt signalling pathway affects mTORC2 and mTORC1. PI3k generates PIP3, PIP3 further activates PDK1, PDK1 activates Akt through phosphorylation, and Akt further activates mTORC2. DHQ has an inhibitory effect on ROS production. After the addition of DHQ, the activity of ROS is reduced, the regulatory effect of ROS on the PI3k pathway is weakened, the expression of Akt, which is downstream of PI3k, is decreased, and the expression of mTORC2, which is downstream of Akt, is inhibited. Akt phosphorylation can regulate GSK3 and reduce cell survival to ameliorate diabetic nephropathy in rats. (Red indicates increase/promotion, green indicates decrease/inhibition, and yellow indicates promotion of the original pathway but inhibition of this pathway.)

Dio is a metabolite found in the petals of *O. fragrans*. It can be obtained through a series of chemical reactions using hesperidin as the precursor compound. Dio is an aglycone of the flavonoid glycoside geraniol (Tang and Zhao, 2014; Ma, et al., 2018). Dio, a natural compound with a molecular formula of C_16_H_12_O_6_, mainly exists in plants in the free form or as a glycoside and is normally a yellow powder at room temperature. Its mechanisms of action in the treatment of different diseases are distinct and need to be analysed according to the specific situation. Studies have found that geraniol has anti-inflammatory, antibacterial, anticancer, and antioxidant properties (Si, et al., 2020). Animal studies have shown that Dio can stimulate MH7A cells and increase the apoptosis rate to affect the activation of the PI3K/Akt and NF-κB signalling pathways, thereby inhibiting the development of myocardial infarction (Chen, et al., 2020c) (Figure 3A). On this basis, Wang et al. further found that geraniol can alleviate isoproterenol-induced symptoms of cardiac hypertrophy in mice, potentially reducing the risk of heart disease (Dong, et al., 2020).

#### Dihydroquercetin

DHQ is an important flavonoid found in nature; Its molecular formula is C_15_H_12_O_7_, and it is generally pale yellow or colourless needle-like crystals, its melting point is 240°C, it is easily soluble in ethanol, acetic acid, boiling water and other solvents. DHQ is slightly soluble in cold water, it is almost insoluble in benzene, and it has no odour. The synthesis of DHQ involves two pathways: the phenylpropane pathway and the flavonoid pathway. Cinnamic acid is generated from phenylalanine by phenylalanine ammonia lyase, and then the generation of coumaric acid from cinnamic acid is catalysed by cinnamic acid hydroxylase. The production of naringenin chalcone from coumaric acid is catalysed by 4-coumarin-CoA ligase and chalcone synthase, and then the production of DHQ from naringenin chalcone is catalysed by chalcone isomerase and flavanone 3-hydroxylase dihydroflavonols. DHQ is found in the petals of *O. fragrans*, and scholars at home and abroad have found that DHQ has antioxidant, antiviral, anti-inflammatory, anti-allergic, anti-apoptotic, and antitumour properties along with other medicinal activities and protects the liver (Wang et al., 2011a; Xue, et al., 2017; Lin, et al., 2022). Oxidation and hyperglycaemia can also be affected by DHQ. Moreover, DHQ lowers blood pressure, alleviates cardiovascular diseases and can also repair brain tissue. and ameliorate brain injury and promote the differentiation of osteoblasts (Wang et al., 2011b). In addition, Ying et al. found that DHQ may reduce the symptoms of diabetic nephropathy in rats by affecting the mTORC2/Akt signalling pathway (Qiao, et al., 2020) (Figure 3B).

#### Astilbin

Astilbin is primarily found in the petals of *O. fragrans*. The molecular formula of astilbin, which exists as a white crystalline powder at room temperature that is easily soluble in methanol and ethanol and slightly soluble in water is C_21_H_22_O_11_. Recent studies have found that astilbin may have antidepressant, antioxidant, antidiabetic, analgesic and antibacterial properties, inhibit oedema, protect the liver and kidney, promote chondrocyte proliferation, and exert anti-inflammatory and immunosuppressive effects (Dan, 2014; Wei, et al., 2021). Li studied the mechanism underlying the protect effect of astilbin against cerebral ischaemia–reperfusion injury and found that it can activate the PI3K-Akt pathway and regulate the MAPK pathway (Yu, 2020). Xu studied the role and mechanism of astilbin in regulating Breg cells in inflammatory bowel disease and found that it can induce human PBMCs and mouse lymphocytes to secrete factors with regulatory effects, thereby suppressing the immune response, inhibiting cell apoptosis and preventing inflammation to alleviate ischaemia–reperfusion injury (Xu, 2020).

#### Luteolin

Luteolin is mostly found in the leaves of *O. fragrans* (Tang and Zhao, 2014). Luteolin is a natural flavonoid, and its molecular formula is C_15_H_10_O_6_. Luteolin usually exists as yellow needle-like crystals, has a melting point of approximately 330°C, and is weakly acidic, soluble in alkaline solution, slightly soluble in water, and stable under normal conditions. It is obtained by demethylation of geraniol via pyridine hydrochloride. It can also be produced by saccharol via flavonoid synthase I and flavonoid synthase II. Studies have found that luteolin has medicinal properties, such as antioxidant and antitumor properties, and that it protects the nervous system and enhances memory (Song, et al., 2015; Fu, et al., 2019). While screening for flavonoids with anti-gout effects, Hao found that luteolin can ameliorate hyperuricaemia and acute gouty arthritis by reducing the levels of inflammatory factors (TNF-α, IL-1β) in rats (Yue, 2019). Yang studied the effect of luteolin on acute liver injury induced by inorganic mercury in mice and found that luteolin can alleviate the symptoms of acute liver injury and anaemia caused by mercury (Yang, 2018).

#### Naringenin

Naringenin is a natural organic compound with a molecular formula of C_15_H_12_O_5_. It exists as a yellow powder and is soluble in ethanol, ether and benzene and almost insoluble in water. It should be stored in a cool, dry place away from light and high temperatures. Naringenin is produced from naringenin chalcone through chalcone isomerase. Naringenin is found not only in *O. fragrans* but also in *Citrus sinensis*, *Pteris cretica, Lycopersicon esculentum* Miller peel, *Citrus aurantium* Linnaeus, *Allium fistulosum* L., *Dendrobium nobile* Lindl*.* and other plants (Wu, et al., 2017; Lin, et al., 2022). According to the literature, naringenin has anti-inflammatory, antibacterial, antioxidant, antitumor, anti-lung damage, antiaging, and anticancer (anti-gastric cancer, anti-liver cancer, anti-breast cancer, anti-colon cancer, etc.) properties and can treat metabolic diseases such as diabetes, hypertension, and hyperlipidaemia (Zeng, et al., 2016; Jing, et al., 2019). Studies have also shown that naringenin can reduce apoptosis by activating the ER and the P13K/Akt signalling pathway (Qi, et al., 2020). β-Amyloid (Aβ25-35) alters the expression of phosphorylated Tau protein/total Tau protein in PC12 cells, thereby enhancing their proliferation. By studying the pharmacological mechanism of naringenin in the treatment of coronary heart disease, Wang and others found that naringenin inhibits coronary atherosclerosis and protects cardiomyocytes (Wang and Wang, 2021). Zeng and others found that naringenin has a similar structure as oestrogen- and can regulate the secondary messengers of downstream signalling pathways (the PKC pathway, AKT pathway, MAPK pathway, etc.) (Zeng, et al., 2018). Thus, naringenin plays a role in immune regulation. It has been used in formulas for the treatment of various diseases, such as Fuzheng Huayu tablets/capsules, Songhuafen *Ganoderma lucidum* tablets, Liujunzi decoction for the treatment of cervical cancer, and Shi Da Gonglao decoction for the treatment of hepatitis (Li et al., 2020b; Li et al., 2021b; Yang and Yang, 2021).

### Terpenoids

*O. fragrans* has a unique fragrance, largely due to the presence of volatile components called terpenoids. *O. fragrans* flowers are used for the production of essential oils and raw materials for various spices. Recently, the medicinal effects of terpenoids in *O. fragrans*, e.g., their antioxidant and lipid-lowering properties, have attracted the attention of researchers (Yue, et al., 2013) (Figure 2B).

#### Loganic Acid

Loganic acid, which has a molecular formula of C_16_H_24_O_10_, is an iridoid glycoside compound that normally exists as a white crystalline powder. It should be stored at a temperature of 2–8°C and protected from light. If it is exposed to air for a long time, its physical and chemical properties are altered. Loganic acid is produced from 7-deoxypolysaccharide, and this reaction is catalysed by 7-deoxyxylan hydroxylase. It is present in the petals of *O. fragrans* (Tang and Zhao, 2014; Lv, 2021). and its medicinal properties primarily include anti-lipogenesis and anti-osteoporosis properties. Park *et al.* studied the effect of loganic acid on the adipogenesis of 3T3-L1 preadipocytes and cells in ovariectomy-induced obesity model mice and found that loganic acid can prevent the adipocyte differentiation of 3T3-L1 preadipocytes and cells in ovariectomized mice by reducing the expression of adipogenesis-inducing genes to exert an antiadipogenic effect (Park, et al., 2018). Park et al. found that loganic acid can increase osteoblast production and upregulated the expression of related genes to induce the differentiation of osteoblasts and prevent the differentiation of primary cultured osteoclasts, indicating that loganic acid may be a potential antiosteoporosis agent (Park, et al., 2020).

#### Oleoside

Oleoside is a natural iridoid glycoside compound with a molecular formula of C_16_H_22_O_11_. It normally exists as a powder and is mostly found in plants in the Oleaceae family. It has also been found in the flowers of *O. fragrans* (Yue, et al., 2013; Tang and Zhao, 2014; Lv, 2021). Oleoside promotes the activity of detoxification enzymes (such as SOD, CAT, GSR, GST, etc.), competes with coenzyme Q as the electron carrier in the mitochondrial electron transport chain, acts as a free radical scavenging antioxidant and inhibits lipid peroxidation to exerts antioxidation effects. Currently, there is little research on oleoside. Most oleosides currently available on the market are oleoside-11-methyl esters. Oleoside-11-methyl esters are used as reference substances in traditional Chinese medicine research and development. Because of the low output and good effect of oleoside-11-methyl esters, they are expensive. In addition, the better the packaging specifications are, the higher the purity and price are, with the price being up to 1400 yuan/5 mg. Oleosides have a very large potential use value, so future research on oleoside compounds is needed (Fei, et al., 2010; Castejón, et al., 2020; Araújo, et al., 2021).

#### Secoxyloganin

Secoxyloganin is found not only in the flowers of *O. fragrans* but also in *Lonicera japonica* Thunb. It is an iridoid compound (Lv, 2021) and has a molecular formula of C_17_H_24_O_11_. It normally exists as a solid and needs to be stored at low temperatures. Secoxyloganin has anti-inflammatory, vasoconstriction, antiviral, antitumour and antibacterial activities (Camargo, et al., 2020). While identifying the sensitizing components in reducing injection by ultrafiltration and high-performance liquid chromatography. Wang et al. found that chlorogenic acid can cause allergic reactions but that it has no obvious sensitizing effect (Wang, et al., 2016). There are few research materials for studying secoxyloganin, and related reagents are mostly used by research institutes and university laboratories. Although the market price of secoxyloganin is lower than that of oleoside-11-methyl, it is still more expensive than other compounds. Therefore, secoxyloganin has potential medicinal value and should be studied in the future scientific.

#### Oleanolic Acid

Oleanolic acid is a pentacyclic triterpenoid compound with a molecular formula of C_30_H_48_O_3_. It mainly exists in plants in the free form, normally exists as white needle-like crystals, and is odourless and tasteless. The melting point of oleanolic acid is approximately 310°C, and it is almost insoluble in water and slightly soluble in ethanol and chloroform. It is unstable in acids and bases. Oleanolic acid is produced from β-aromatic resin by oleanolic acid synthase and is mostly found in the flowers and fruits of *O. fragrans* (Wu, et al., 2017). Studies have found that oleanolic acid has antitumor effects, protects the liver, lowers enzyme activity, strengthens the heart, promotes liver cell regeneration, exerts anti-inflammatory effects, promotes lymphocyte proliferation, lowers blood lipid and blood sugar levels, inhibits platelet aggregation, and protects chromosomes from damage. In addition, studies have found that free oleanolic acid can scavenge DPPH free radicals and protect human umbilical vein endothelial cells from oxidative damage. Moreover, oleanolic acid has anticancer effects; for example, it can cause apoptosis of oesophageal cancer cells through the regulation of signalling pathways, and it has few adverse reactions and low toxicity. Therefore, this compound is a good pharmaceutical agent (Shen, et al., 2019; Li et al., 2021a; Zhang, et al., 2021).

#### Maslinic Acid

Maslinic acid is mostly found in the flowers and fruits of *O. fragrans* (Yang, et al., 2021). Maslinic acid is also a pentacyclic triterpenoid with a molecular formula of C_30_H_48_O_4_, and it usually exists as a light yellow powder. This compound lowers blood sugar levels, has anti-inflammatory and antifibrotic effects, and inhibits parasitic infections. It is effective in treating colon cancer, colorectal cancer, pancreatic cancer, lung cancer, etc. (Li, et al., 2017; Xuan, et al., 2021; Yang, et al., 2021). By studying the effects of maslinic acid on H_2_O_2_-induced oxidative damage to BRL-3A cells and the p38 and Nrf2/HO-1 pathways, Li *et al.* found that maslinic acid can alleviate oxidative damage induced by hydrogen peroxide (Li et al., 2020a). Hu et al. found that maslinic acid has a dose-dependent effect on the proliferation of HeLa cervical cancer cells; that is, the higher the dose of maslinic acid is, the poorer the proliferation ability of HeLa cells (Hu, et al., 2021).

### Phenolic Acid Compounds

Many literature reviews have indicated that phenolic acid compounds, which are slightly less researched than terpenoids and flavonoids but are still important, are also present in *O. fragrans*. Most phenolic acid compounds have unstable structures and are easily affected by external biological, physical and chemical factors (Omar, 2010; Lin, et al., 2017; Ju, et al., 2019). They are also allelochemicals that interact with crops and are widely distributed in nature. The nature and concentration of phenolic acid compounds in different plant tissues are very different, and they have high medicinal value. For example, they can inhibit the proliferation, invasion and metastasis of liver cancer cells; promote the autophagy of liver cancer cells; exert anti-inflammatory, antioxidant, anti-allergic, antitumour, and antibacterial effects; and protect blood vessels (Hao, et al., 2017) (Figure 2C).

#### Tyrosol

The molecular formula of Ty is C_8_H_10_O_2_, and Ty normally exists as a yellow–green fine powder. Ty, which is a derivative of phenyl ethyl alcohol, is present in the flowers of *O. fragrans*. The precursor of Ty is generated from tyrosine by aromatic aldehyde synthase, and the Ty precursor is further synthesized into Ty by other enzymes (Wu, et al., 2017; Yang, et al., 2021). Ty is a mild compound that has a strong ability to scavenge highly toxic hydroxyl free radicals and can prevent the occurrence of coronary heart disease and tumours (García-Villalba, et al., 2015). Ty also has antioxidative, anti-inflammatory and antibacterial effects; lowers blood sugar levels; and increases longevity. It also has a therapeutic on common neurological diseases, such as Alzheimer’s disease and Parkinson’s disease. Studies have shown that the Ty content in the plasma reaches the peak level 2 h after ingestion, indicating that Ty conjugates are a major metabolite in the plasma (Shi, et al., 2020). Furthermore, Ty can promote the proliferation of endothelial cells (He, et al., 2017).

#### p-Hydroxyphenylacetic Acid

The molecular formula of p-hydroxyphenylacetic acid is C_8_H_8_O_3_, and it is normally exists as a white crystalline powder. It is slightly soluble in water and soluble in ether, ethanol and ethyl acetate. It should be stored in a cool dry place, and direct contact with the skin and eyes should be avoided during use. P-Hydroxyphenylacetic acid can be obtained through a series of chemical reactions between phenol and glyoxylic acid under alkaline conditions. It is an important rejuvenating herb. This compound is also present in many medicinal plants, such as *Forsythia suspensa*, *Taraxacum mongolicum* Hand.-Mazz, *Rhodiola rosea* L., *Cedrus deodara* (Roxb.) G. Don, and *Pistacia chinensis* Bunge, and a previous study reported that *O. fragrans* flowers contain this compound (Wei, et al., 2015; Wu, et al., 2017). P-Hydroxyphenyl acetic acid is also a raw material used to synthesize the anti-Parkinsonian drug pimavanserin (Hu, et al., 2020). Furthermore, it can also be used to synthesize the cardiovascular drug atenolol, which is used to treat various side effects caused by hypertension, and the analgesic and anti-inflammatory drug ibuprofen. It can even be used to synthesize the antibiotic drug laoxycephalosporin, which is used to alleviate the symptoms of antibiotic-sensitive bacterial infections, such as sepsis, bronchitis, cholecystitis and other types of inflammation (Peng, et al., 2014; Bai, 2016).

#### Rosmarinic Acid

RA is a water-soluble natural phenolic acid compound with a molecular formula of C_18_H_16_O_8_. It is a natural antioxidant with strong antioxidant activity. At low concentrations, it exists as a light yellow to brown powder, and at high concentrations, it exists as a white powder. It is more suitable for storage under acidic and low-temperature conditions. Since light has a strong impact on RA, it should be protected from light as much as possible during use. RA is produced from phenylalanine through a reaction catalysed by a series of enzymes, such as phenylalanine lyase and RA synthase, and is mostly found in the flowers of *O. fragrans* (Xue, et al., 2018; Lv, 2021). The currently recognized pharmacological activities of RA and its derivatives include antimicrobial (bacteria, fungi, viruses), anti-inflammatory (dermatitis, pneumonia, nephritis, periodontitis, arthritis, etc.), antitumour, antidepressant, and antioxidant properties. It can also prevent and treat allergic rhinitis induced by PM2.5 and inhibit the formation of kidney stones (Ke and Min, 2019; Zhou, et al., 2021). Lucia Cattaneo et al. found that rosemary extract inhibits the proliferation of A375 human melanoma cells and may induce apoptosis of colorectal cancer cells by interfering with the MAPK/ERK pathway (Cattaneo, et al., 2015).

#### Salicylic Acid

SA is a fat-soluble compound with a chemical formula of C_7_H_6_O_3_. It generally exists as a white needle-like crystal or hairy crystalline powder and has a melting point of approximately 159°C. It is stable at room temperature and as it has a corrosive effect, it can irritate the skin and mucous membranes. The main biosynthetic pathway of SA is the isochorismate synthase pathway, which is catalysed by chorismate synthase and isochorismate lyase, and SA is present in the flowers of *O. fragrans* (Zhang, 2008; Wang, et al., 2014). SA is widely used in many industries, specifically the pharmaceutical industry for the preparation of various drugs, such as aspirin, Zhitongling, sodium salicylate, and salicylamide. It is also used in the diagnosis and treatment of papules and pustular rosacea and can improve skin texture, and high concentrations of SA can exfoliate keratinocytes to achieve whitening; thus, it is often used to treat hair follicle sebaceous gland diseases (Cockayne, et al., 2011; Dayal, et al., 2020). SA can also prevent thrombosis, reduce blood viscosity, inhibit platelet aggregation and adhesion, reverse the hardening of blood vessels, alter vascular permeability, prevent arteriosclerosis, etc. (Cockayne, et al., 2011; Xin, et al., 2018; Ting, et al., 2021).

#### Protocatechualdehyde

The molecular formula of PCA is C_7_H_6_O_3_, and PCA exists as a light beige needle crystal or off-white powder. PCA has a melting point of 153°C and is soluble in cold water; easily soluble in ethanol, acetone, ethyl acetate, ether and hot water; and insoluble in benzene and chloroform. PCA can be prepared from p-hydroxybenzaldehyde through bromination, methoxylation, hydrolysis and other reactions, and it is mostly found in *O. fragrans* (Zhang, 2008; Xue, et al., 2018). Its pharmacological activity is relatively extensive, but studies have shown that it has some degree of toxicity. Thus, it is important to be careful when using this compound. PCA is known to inhibit inflammation, cell apoptosis, platelet aggregation, leukocyte chemotaxis and migration; exert antioxidative effects and effects against liver fibrosis, hepatitis B virus infection, and septicemia; promote microcirculation; reduces morphological abnormalities in acanthocytes; exert neuroprotection; hinder the formation of melanin; promote bone healing; and protect the myocardium by reducing calcium overload. It can also protect against cerebral ischaemia in rats by downregulating GFAP and AQP-4 protein expression in brain tissue when reperfusion injury does not disrupt neurovascular unit homeostasis (Zhang, et al., 2013; Jin, et al., 2021).

## Conclusion and Prospects

*O. fragrans* is widely cultivated in China because it is beautiful and easy to grow. In addition, it is a relatively common tree species. It is mostly used as a street tree for ornamental purposes, and research on its use as a medicinal material is relatively limited. Many years ago, the pharmacological effects of *O. fragrans* was investigated, and this plant was used to treat diseases. At that time, people did not know what metabolites were, and metabolomics techniques were not available; however, researchers used observation and experimentation to identify the medicinal properties of various plants to eradicate disease sources. With advances in science and technology, the therapeutic efficacy of *O. fragrans* in clinical trials should be studied. If more human and financial resources are invested in scientific research in the future, this plant may also be used as a raw material for medicinal agents. The secondary metabolites mentioned in this article mainly have antioxidative, anticancer, anti-inflammatory and antibacterial activities (Table 2). However, more research is needed on the other pharmacological activities of these metabolites. While consulting the relevant literature on representative metabolites of *O. fragrans*, we found that the understanding of the synthetic pathways and mechanisms of these plants metabolites is incomplete, and more research on this topic is also needed. Therefore, we need to spend more time researching the metabolites of *O. fragrans*. At present, advanced molecular biotechnology methods, such as variable splicing and SWATH-MS technology, have been applied to study woody plants (Chen, et al., 2020a; Chen, et al., 2021b). Alternative splicing is an important transcriptional regulation mechanism through which we can control plant growth and development. For example, the colour of *O. fragrans* can be altered by artificially moving the start sites of certain genes (Chen, et al., 2020b; Zhang, et al., 2020; Song et al., 2021b). Proteomic analysis based on SWATH-MS can help better determine the mechanism of action of plants (Chen, et al., 2021a; Shen, et al., 2021), and we can apply these advanced technologies in future research on *O. fragrans*. Whole *O. fragrans* can be used as a medicine and is a traditional Chinese medicinal material that has been used for many years. Metabolomics, which does not require much effort, has been developed as a novel technique in recent years. However, we need to find a way to better combine metabolomics data with data on the pharmacological effects of *O. fragrans*. We hope that in the future, research will focus on the development of *O. fragrans* as a medicinal agent for treating different diseases.

**TABLE 2:** Medicinal efficacy of major secondary metabolites in *Osmanthus fragrans*.

Metabolites	Location	Function
Geraniol	Flower	Anti-inflammatory, anti-cancer, anti-oxidant
Dihydroquercetin	Flower	Anti-virus, anti-apoptosis, anti-tumor
Astilbin	Flower	Anti-depressant, anti-diabetic, analgesic and antibacterial
Luteolin	Leaves	Anti-tumor, protect nervous system, enhance memory
Naringenin	Flower	Anti-inflammatory, anti-bacterial, anti-lung damage, anti-aging
Loganic acid	Flower	Anti-lipogenesis, anti-osteoporosis
Oleoside	Flower	Anti-oxidation
Serotonin	Flower	Antiviral, antitumor and antibacterial activity
Oleanolic acid	Flower, Seeds	Protect liver, lower enzymes, strengthen heart, lower blood fat
Maslinic acid	Flower	Anti-inflammatory, anti-fibrosis
Tyrosol	Flower	Prevent coronary heart disease and tumors, anti-oxidative stress
P-hydroxyphenylacetic acid	Flower	Analgesic and anti-inflammatory
Rosmarinic acid	Flower	Anti-microbial, anti-oxidant, inhibit kidney stones
Salicylic acid	Flower	Soften blood vessels and treat skin diseases
Protocatechualdehyde	Flower	Inhibit cell apoptosis, anti-septicemia
Ursolic acid	Seeds	Antitumor
Salidroside	Seeds	Lower blood sugar, anti-oxidation, anti-inflammatory
Linoleic acid	Seeds	Anti-oxidation
Melanin	Flower	Anti-oxidant, anti-aging
Palmitic acid	Flower	Anti-oxidation

The table summarizes the structure and function of the 15 compounds in this paper and introduces the structure, function and medicinal value of secondary metabolites that do not appear but also come from Osmanthus fragrans.

## Abbreviations

D-GalN, D-galactosamine; DHQ, dihydroquercetin; Dio, diosmetin; DN, diabetic nephropathy; O. fragrans, Osmanthus fragrans; PCA, protocatechualdehyde; RA, rosmarinic acid; ROS, reactive oxygen species; SA, salicylic acid; Ty, tyrosol.
